# Determination of Moisture and Protein Content in Living Mealworm Larvae (*Tenebrio molitor* L.) Using Near-Infrared Reflectance Spectroscopy (NIRS)

**DOI:** 10.3390/insects13060560

**Published:** 2022-06-20

**Authors:** Nina Kröncke, Rainer Benning

**Affiliations:** Institute of Food Technology and Bioprocess Engineering, University of Applied Sciences Bremerhaven, An der Karlstadt 8, 27568 Bremerhaven, Germany; rbenning@hs-bremerhaven.de

**Keywords:** *Tenebrio molitor*, edible insects, near-infrared spectroscopy, nutritional composition, chemometrics, rapid method, water supply

## Abstract

**Simple Summary:**

Insects are increasingly becoming a new protein source for animal feed and human food. Spectroscopic methods, such as near-infrared reflectance spectroscopy, represent a non-destructive and rapid technique that can be applied to perform an online analysis in chemical composition. The aim of the research was to determine the moisture and protein content of living mealworm larvae using near-infrared spectroscopy as a new technique in analyzing nutritional changes. The prediction results of the near-infrared reflectance measurements of living mealworm larvae are presented in this study. The moisture and protein content of the larvae could be predicted with high accuracy and were specifically manipulated by using different water sources (pure water and carrots) and amounts and varying humidity. It was also determined that the larvae can be optimally provided with pure water as well as carrots. High humidity led to faster growth and a higher final weight, which has a positive effect on reducing the time to harvest. This study can help insect producers to have the possibility to measure the composition of the larvae quickly and easily using near-infrared spectroscopy, modify larval composition with regard to water and protein content and improve rearing conditions in terms of water supply for mealworm larvae.

**Abstract:**

Yellow mealworm larvae (*Tenebrio molitor* L.) are a sustainable source of protein for food and feed. This study represents a new approach in analyzing changes in the nutritional composition of mealworm larvae using near-infrared reflectance spectroscopy (NIRS) combined with multivariate analysis. The moisture and protein content of living larvae were scanned with a near-infrared spectrometer using wavelengths from 1100 to 2100 nm. Different feeding groups with varying moisture sources and amount and the difference between low (50%) and high (75%) humidity were tested, and the influence on larval moisture and protein content was measured. A calibration was developed, with modified partial least squares as the regression method. The NIR spectra were influenced by the moisture and protein content of the larvae, because the absorbance values of the larval groups differed greatly. The coefficient of the determination of calibration (R^2^_c_) and prediction (R^2^_p_) were over 0.98 for moisture and over 0.94 for protein content. The moisture source and content also had a significant influence on the weight gain of the larvae. Consequently, significant differences in protein content could be determined, depending on the water supply available. With respect to wet weight, the larvae moisture content varied from 60 to 74% and protein content from 16 to 24%. This investigation revealed that with non-invasive NIRS online monitoring, the composition of insects can be continuously recorded and evaluated so that specific feeding can be carried out in the course of larval development and composition.

## 1. Introduction

Numerous forecasts indicate that the world population will reach over 9 billion people by 2050 [1], which will require roughly double the current food production [2]. Global warming is gradually reducing the areas available for food production worldwide [3]. Climate change and environmental degradation also have a negative impact on food productivity [4]. Regarding the increasing scarcity of resources, several alternative protein sources are proposed, with insects receiving the most attention [5]. The most promising and well-studied edible insects for industrial feed production are the black soldier fly (*Hermetia illucens*), yellow mealworm (*Tenebrio molitor*), locusts (*Locusta migratoria*) and termites [3]. The yellow mealworm is, due to the frequency of consumption, a promising candidate for breeding and production on an industrial scale [4]. *Tenebrio molitor* is the first insect species that has been declared safe as food and feed according to the Scientific Opinion of the European Food Safety Authority (EFSA) on edible insects in January 2021. Insects have a lower environmental impact (e.g., land and water requirements, greenhouse gas emissions) compared to livestock such as pigs or chickens, and indoor, urban and vertical farming are advantageous for insect rearing [6]. Insects have high water, protein and fat content as well as polyunsaturated fatty acids, vitamins and minerals and are considered an alternative to ensure feed and food security [2,4]. Protein content can range between 25 and 75% (dry matter basis) [7,8,9,10,11] and also the fat content has a high variance with 10 to 70% on a dry matter basis [8,11,12,13]. *T. molitor* larvae need a source of water for optimal growth, even if they have a high level of protection against evaporation [14]. They also have high growth rates and feed conversion efficiencies because they do not need energy to maintain a constant body temperature. Insects can be reared on organic side streams and transformed into high-value feed and food resources [4,15,16]. Several studies showed that the nutritional composition of the feeding substrate has an influence on insect larval composition [7,17,18,19], and relative humidity also has an impact on the survival, growth rate and life cycle of *Tenebrio molitor*. Humidity over 70% showed the best results for larval development, survival and growth [20,21]. In order to ensure balanced and targeted feeding or to achieve a desired larval composition, it is essential to monitor and regulate larval feeding and water supply.

Near-infrared reflectance spectroscopy (NIRS) emerged as a fast technique for the quantitative determination of several nutritional parameters (i.e., water and protein) simultaneously and is used for quality and process control of food and feed. Current standard analytical methods such as the Kjeldahl method for protein analysis are extremely inefficient in terms of resources and time, and they are completely unsuitable for small insect producers. Therefore, NIRS offers a high potential to minimize costs and time concerning analytical investigations of insect samples. Consequently, it often replaces standard wet-chemical procedures for quality and process control as the measurement is non-destructive and can be carried out without complex sample preparation [22]. As an optical method, it is based on the interaction of light with the sample and has been one of the most successful techniques used for food quality assessment [23]. Many studies showed the correlation between chemical composition and corresponding absorbance spectra recorded by near-infrared spectroscopy [24,25]. NIR spectra result from overtones and the combination bonds of C-H, O-H and N-H groups of stretching vibrations [26,27], which are the most abundant chemical constituents in biological samples. NIRS has been successfully used for the analysis of water and protein by different food sectors, such as meat [27,28,29], cereals [30,31], fruit [32,33] and cheese [34,35]. There are also some applications that could be used for the detection of insect pests in stored grains [36,37,38], but without analyzing the chemical composition of these insects. It is also possible to analyze the protein content of insect-based energy bars [39]. Some NIR models were built to classify edible insect powders using Fourier-transform infrared spectroscopy (FTIR) [40]. There is currently no known application for determining the moisture and protein content of living insect larvae by near-infrared reflectance spectroscopy.

The objective of the present work was to obtain NIR calibrations and develop multivariate classification models to predict the water and protein content in living mealworm larvae. We also wanted to find out the best way in which the larvae can be supplied with water to ensure optimal growth for commercial rearing and whether it is possible to obtain diverse reference data for the moisture and protein content of the larvae by offering different moisture sources and volumes.

## 2. Materials and Methods

### 2.1. Insect Samples and Feeding Groups

Larvae of *Tenebrio molitor* used in this study were cultured at the University of Applied Sciences Bremerhaven (Bremerhaven, Germany). Larvae were kept under controlled conditions in a constant climate chamber (HPP 110, Memmert, Schwabach, Germany) at a temperature of 27 °C with a relative humidity of 70% and were fed ad libitum with wheat bran. Larvae for the experiment were selected when they reached eight weeks. Each feeding group contained 100 individuals, with an average weight of 12.04 ± 0.01 mg per individual at the start of the experiment, and was fed ad libitum with wheat bran at a rearing temperature of 27 °C. Relative humidity was varied (50 and 75%), as was the water source (none, pure water or carrots), water volume and watering interval (daily, every two days, weekly) in order to modify larval composition and investigate the influence on the biomass growth. Table 1 presents the different feeding groups. Carrots were analyzed for their water content (average water content of 88%), resulting in a water content of about 2.6 g per 3.0 g carrot. Consequently, the amount of pure water was adjusted accordingly and spread evenly with a spray flask on the wheat bran. Larvae were kept in 400 mL glass beakers for a five-week period.

After an experimental period of five weeks, larvae were separated from food leftovers and frass, starved for 24 h and measured using NIRS. Larval weight, survival rate and the amount of food leftovers and frass were also recorded. Following parameters were then calculated as described by Waldbauer (1968) [41]:

Data for food consumption and weight gain per larvae (LWGpL; Equation (1)) were calculated and used for determination of feed utilization parameters:

(1) $$\mathrm{LWGpL}=\frac{\mathrm{Larval}\;\mathrm{weight}\;\mathrm{end}\;-\;\mathrm{Larval}\;\mathrm{weight}\;\mathrm{start}\;}{\mathrm{Number}\;\mathrm{of}\;\mathrm{larvae}\;\mathrm{at}\;\mathrm{beginning}\;-\;\mathrm{Number}\;\mathrm{of}\;\mathrm{dead}\;\mathrm{larvae}}$$

The feed conversion ratio (FCR; Equation (2)) as the amount of feed needed (in kg) to obtain one kg of wet weight increase in the production animal:

(2) $$\mathrm{FCR}=\frac{\mathrm{Feed}\;\mathrm{consumed}}{\mathrm{Weight}\;\mathrm{gained}}$$

The efficiency of conversion of ingested food (ECI, %; Equation (3)) was calculated according to the following formula:

(3) $$\mathrm{ECI}=\frac{\mathrm{Weight}\;\mathrm{gained}}{\mathrm{Feed}\;\mathrm{consumed}}\;\times \;100\%$$

as well as the specific growth rate (SGR, %/day; Equation (4)):

(4) $$\mathrm{SGR}=\frac{\mathrm{lnFBW}-\;\mathrm{lnIBW}}{\mathrm{experimental}\;\mathrm{days}}\;\times \;100\%$$

where FBW stands for final body weight and IBW for initial body weight. After recording the data, larvae were frozen at −21 °C for 48 h in a freezer (HAS 47520, Beko, Neu-Isenburg, Germany) and stored prior to performing chemical analyses.

### 2.2. Spectra Collection

Living mealworm larvae were placed in a glass petri dish with an internal diameter of 70 mm and a depth of 20 mm. Spectra were scanned and collected using a near-infrared reflectance spectrometer (PSS 2120, Polytec GmbH, Waldbronn, Germany) with a wavelength between 1100 and 2100 nm. All samples were scanned ten times and the spectra were averaged (50 spectra in total per sample) prior to conducting statistical analyses. 

### 2.3. Multivariate Analysis

Spectral data were prepared using Matlab (version R2020a, The MathWorks Inc., Natick, MA, USA) combined with PLS Toolbox (version 8.9.1, Eigenvector Research Inc., Wenatchee, WA, USA) to develop a calibration model for the prediction of moisture and protein content of living mealworm larvae. Samples were randomly partitioned into two subsets, a calibration set (*n* = 120) and an independent validation set (*n* = 60), to develop the prediction equations. Partial least squares (PLS) regression was used to obtain classification models in order to discriminate the differences in moisture and protein content. Different mathematical methods, such as mean center (MC), multiple scatter correction (MSC) and 1st (1D)- and 2nd (2D)-order derivative, were used for preprocessing. The optimum number of latent variables (LV) was defined at the maximum value of the variance. The predictive capability and accuracy of the PLS models were evaluated based on the coefficient of determination for calibration (R^2^_c_), root mean square error of calibration (RMSEC), coefficient of determination in prediction (R^2^_p_) and root mean square error of prediction (RMSEP). The ratio of performance to deviation (RPD), which is defined as standard deviation/RMSEP, was calculated to determine the practical utility applicability of the prediction models. High RPD values are required, whereas RPD values above 10 are considered equal to the reference method, RPD values greater than 3 are regarded as adequate for routine analysis and RPD higher than 2 represents a robust calibration. Where RPD values below 1.5, the results are unreliable and models have no prediction ability [22,42,43]. The preferred model was chosen with respect to the highest RPD and R^2^_p_, the lowest RMSEP, and number of latent variables were limited (≤10).

### 2.4. Proximate Analysis

Nutritional composition of *Tenebrio molitor* larvae was analyzed as described previously [44]. Moisture content was determined by drying larvae at 103 °C for 4 h in a drying oven (U10, Memmert, Schwabach, Germany). Protein content was measured using the Kjeldahl method and calculated according to DIN EN 25663 and the Association of German Agricultural Analytic and Research Institutes by multiplying the measured nitrogen content with a factor of 6.25 [45]. Protein contents were expressed as a percentage on a fresh weight basis.

### 2.5. Statistical Analysis

Statistical significance of the feeding results, including analyses, were replicated and performed three times (*n* = 3) and checked for normality and homogeneity of variances. Statistical analysis was performed by one-way ANOVA, followed by a Tukey–Kramer post hoc test using SigmaPlot 12.5 (Systat Software Inc., Düsseldorf, Germany). A confidence interval of 95% (*p* < 0.05) was presumed. 

## 3. Results

### 3.1. Growth Rate and Feed Conversion Efficiency

The larval growth, feed conversion ratio as well as efficiency and specific growth rate are presented in Table 2. The larval weight gain ranged from 8.5 to 60.5 mg per larvae, with the significantly lowest weight gain in the groups without a water source (groups A and C). The larvae mass increased with humidity (75%), so that the larvae were approximately twice as heavy as those reared at 50%, even when no water source was available. All parameters measured were significantly affected by diet type. Dietary supplementation of water and carrots improved the growth rates to 5.1% per day (group K). In the absence of a water source, the growth rates are very low (1.5 and 2.4 ± 0.1 in groups A and C). As the water supply decreases, so does the weight of the food consumed. Furthermore, the increase in body weight per unit of food consumed is greatly reduced. When humidity is at its lowest, the amount of food consumed becomes exceedingly small, and although copious amounts of water may be drunk, the larvae cannot utilize their food well. High humidity of 75% leads to a better growth rate, feed conversion ratio and efficiency. The survival rate in all groups was 100%. 

### 3.2. Nutritional Analysis

The moisture and protein content of the *T. molitor* larvae are shown in Table 3. The moisture content of the *T. molitor* larvae varied between 60.8 ± 0.1 and 72.9 ± 0.5% and was significantly lower (*p* < 0.05) in groups A and C which had no water source. The highest moisture content in the larvae was attained in the groups that had pure water (2.6 g) or carrots (3.0 g) available on a daily basis or pure water (2.6 g) every two days, but there were no significant differences. Increasing the amount of carrot to 10 g did not result in a significant increase in the moisture content. The protein content did not vary as widely as the moisture content, but it was between 16.0 ± 0.6 and 23.5 ± 0.1%. The highest protein content was achieved by group C (50% rh, no water) with 23.5 ± 0.1%, followed by group A (75% rh, no water) with a protein content of 22.1 ± 0.1%. Both groups recorded the lowest moisture content with 60.8 ± 0.1% (C) and 62.7 ± 0.7% (A). On average, the protein content decreases with increasing moisture content.

### 3.3. NIR Spectra and Reference Data

**Figure 1:**
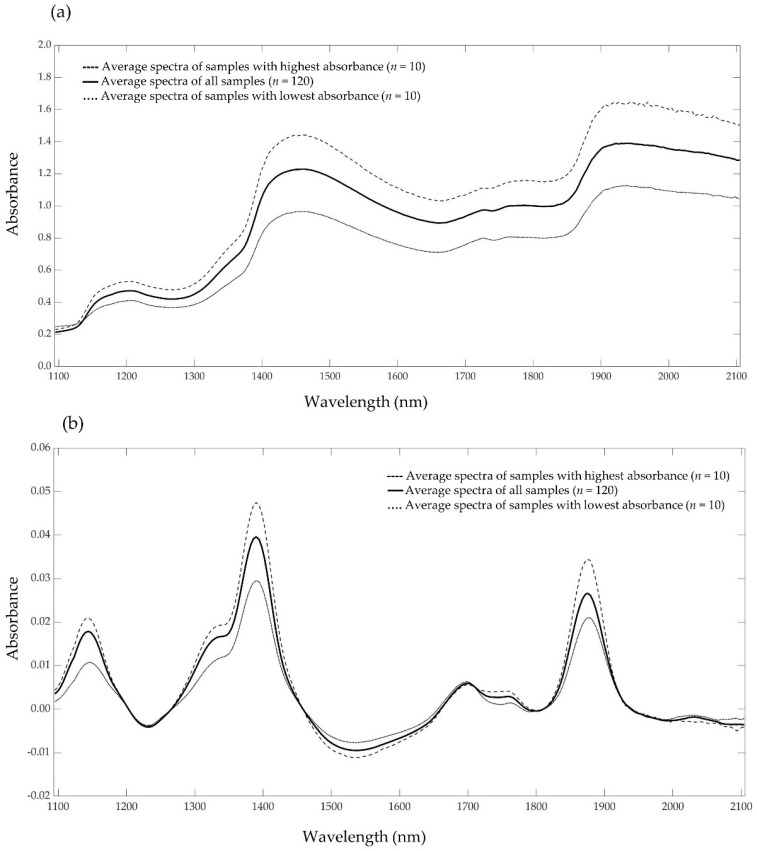
Average NIR raw spectra (**a**) and preprocessed spectra (1st derivative) (**b**) of living mealworm larvae from samples with highest and lowest absorbance compared to all samples.

The average NIR raw spectra and first-order derivative preprocessed spectra are shown in Figure 1 (for further details of the NIR raw and preprocessed data of all groups, see Supplementary Figure S1). In the raw spectra (Figure 1a), six peaks can be identified at wavelengths of about 1200, 1450, 1726, 1785, 1950 and 2050 nm. Characteristic bands around 1200, 1726 and 1785 nm can be attributed to fat content [46,47]. Absorption bands at 1450 and 1950 nm can be related to moisture content [47]. Fluctuations in protein molecules begin at a wavelength around 2050 nm and fat molecules between 1725 and 1739 nm [48]. The first-order derivative preprocessing of the NIR spectra (Figure 1b) showed more abundant spectra bands and pronounced variance. The spectral shape was transformed and the signals highlighted by the first-order derivative attributable to the moisture and protein content of the samples [14,27].

Table 4 outlines the descriptive statistics for the moisture and protein content of the living mealworm larvae in the calibration and validation sets, measured by traditional chemical methods. Data showed a wide range of variability in the moisture content (between 60.4 and 72.9%), which is beneficial for the calibration development [27]. This variability is based on the different water source and humidity for the mealworm larvae. The variability in the protein content was lower, suggesting that the water source and amount does not have such a large impact on the protein composition of the larvae. During the development of the prediction equality, no outliers were detected.

### 3.4. Prediction of Moisture and Protein Content

Table 5 summarizes the performance of the PLS models in the calibration and validation sets, based on raw spectra using different mathematical preprocessing treatments. Calibration models without pre-treatment do not provide the highest prediction accuracy. The calibration models were not improved by using MC, and the number of latent variables could not be reduced. After applying MSC, the predictability of the calibration model enhanced for all parameters and led to a smaller number of latent factors. The combination of MC and MSC with the 1st and 2nd derivative resulted in the smallest number of latent variables but reduced the predictability of the model. However, the mathematical treatment using the first derivative gave the best calibration models on all parameters for the prediction of the moisture and protein content, with high RPD values and very few latent variables.

**Figure 2:**
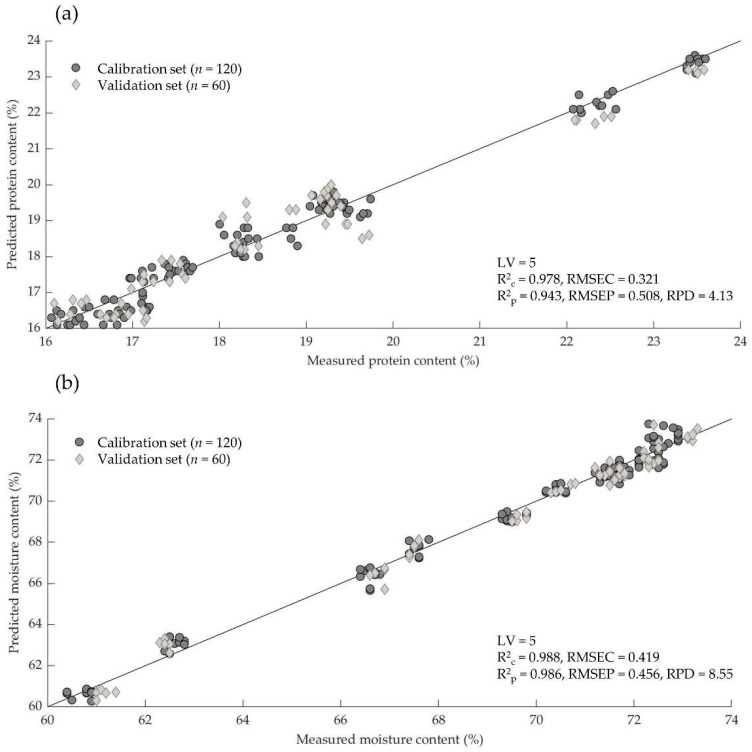
Comparison of measured and predicted values of protein (**a**) and moisture (**b**) content of living mealworm larvae in calibration and validation sets by NIRS (1100–2100 nm).

The best model was selected in terms of a high R^2^_p_ and RPD, a low RMSEP and a limitation of the number of factors (<10). The performance of the selected PLS model in terms of measured versus predicted values of moisture and protein is shown in Figure 2. The statistics indicate the ability to predict the moisture and protein content in living mealworm larvae (Table 5 and Figure 2). However, a mathematical preprocessing of the raw spectra is necessary to increase the prediction accuracy of the NIR models. For the prediction of the protein content (Figure 2a), the best results were observed with the 1st derivative pretreatment (R^2^_p_ = 0.943, RMSEP = 0.508, RPD = 4.13).

For the moisture content, the prediction equation proved to be very reliable in the present study (Figure 2b). The best predictive ability was also achieved with the 1st derivative (R^2^_p_ = 0.986, RMSEP = 0.456, RPD = 8.55). The range of the reference data for moisture is higher than for the protein content, so the prediction accuracy is better for this model too.

Insect breeders can determine larval composition using the two models. However, the requirement for this is a near-infrared reflectance spectrometer, which records the composition of the larvae and links it to the existing calibration for the evaluation of the measured data. The raw data of the calibration for the prediction of the water and protein content of yellow mealworm larvae determined in this study can be made available on request.

## 4. Discussion

The moisture and protein content for all groups of mealworm larvae in this study are comparable to other raw insects [49,50]. Our results clearly demonstrate that the protein content is hugely affected by water source and volume, and the moisture content of the larvae. In the groups with the simultaneous addition of water and carrots, no significant increase in the moisture content of the larvae could be observed, although the volume of water and the addition of carrots were increased. This could be due to the fact that the larvae are saturated with water at some point, meaning there is no advantage in adding more water. For industrial rearing, this points to the fact that mealworm larvae do not need to be watered daily, so the interval can be extended to every two days to achieve acceptable growth.

The results show that the relative humidity has a significant impact on larval composition and the biomass of the individual larvae, but it does not affect the survival rate at optimal rearing temperatures. This was also observed in other studies [20,21]. Larvae reared on dry wheat bran grew much more rapidly when pure water or carrots were available than when they were not, and the larvae showed highly significant differences in their specific growth rates. This is in agreement with the study by Urs and Hopkins [51]. Therefore, for an industrial mass production of *Tenebrio molitor* larvae, an increased humidity of 75% with an addition of a water source such as carrots would presumably lead to a faster growth rate with a higher final weight, which conclusively means a higher yield.

The reduced weight in the groups with no addition of water could also be an indication that evaporation is taking place. Weight loss rarely provides a reliable estimate of evaporation, as fecal excretion and larval metabolism also contribute to the reduced total weight [52]. However, some studies substantiate the conclusion that mealworms are an exception to this principle due to the storage of excreted nitrogen as uric acid and their special metabolism, because metabolic losses are compensated for by the amount of metabolic water gained [53,54]. 

No studies are currently available to predict the moisture and protein content in mealworm larvae or other insects. However, there is a large number of studies on animal products such as meat using NIRS, which are used for comparison. Our results are consistent with previously published data in poultry and beef samples (RPD > 2.56) [55,56,57,58]. On the other hand, no good correlations between the protein reference data and the NIR spectra (RPD < 2.35) for beef [59,60], poultry [61] and lamb [62] could be found in other studies. An explanation may be the analytical differences between NIRS (measures protein) and the Kjeldahl determination (measures nitrogen) [63], and the narrow variation range of protein content in beef samples [29]. *Tenebrio molitor* larvae have a high protein content [10,12,50], but also contain chitin in their cuticle [64]. Therefore, the true protein levels are overestimated when using the nitrogen-to-protein conversion factor of 6.25, due to the presence of non-protein nitrogen from chitin [65]. In our study, we did not determine the chitin content of the larvae and excluded that from the NIR spectra, so chitin and other nitrogen-containing compounds are also reflected. But other studies showed that is it possible to detect raw chitin using NIRS and create a calibration with a high prediction accuracy [66,67]. Hence, the prediction of chitin in living mealworm larvae could be the research topic of next studies. The prediction accuracy of the protein content is good in our study but may be improved by having a broader variability in the reference data, which positively influences the NIRS predictability. The difficulty is also in the measurement of living mealworm larvae, because the protein content can be predicted more precisely in homogenized samples, such as insect flour [68], than in intact samples [56]. The results show that calibration models without pre-treatment do not provide the highest prediction accuracy. This is in agreement with other studies [69,70]. The results for the prediction of the moisture content are much better as compared to beef [56,60], pork [58] or lamb [55]. Additionally, moisture and fat content can be highly correlated, meaning the ability of NIRS to predict moisture is similar to predicting fat content. We therefore wish to work out the correlation of the reference data and NIRS for fat in further studies and create NIR calibration models for the prediction of the fat content in living mealworm larvae.

## 5. Conclusions

The results of the current study prove the possibility of near-infrared reflectance spectroscopy to forecast moisture and protein content in living *Tenebrio molitor* larvae. Near-infrared spectroscopy offers an alternative to the standard analytical methods used to measure the chemical composition of mealworm larvae. It is possible to analyze the samples in their original live form, so that no larvae have to be killed for measurement. We were also able to show that mealworm larvae need a water source for rapid growth. This can be done as pure water or as a vegetable, such as carrots. In addition, it can be advantageous for industrial rearing to increase the humidity to 75%, so that the larvae also tend to grow faster and the time until harvest can be shortened.

## Figures and Tables

**Table 1:** Feeding groups of *T. molitor* larvae.

Group	Relative Humidity (%)	Water Source	Watering Interval
A	75	None	-
B	75	Pure water (2.6 g)	1 × per day
C	50	None	-
D	50	Pure water (2.6 g)	1 × per day
E	50	Carrots (3.0 g)	1 × per week
F	75	Pure water (2.6 g)	Every two days
G	75	Carrots (3.0 g)	1 × per week
H	75	Carrots (3.0 g)	Every two days
I	75	Carrots (3.0 g)	1 × per day
J	75	Carrots (10.0 g)	1 × per week
K	75	Pure water (2.6 g) and carrots (3.0 g)	1 × per week and 1 × per day
L	75	Pure water (2.6 g) and carrots (10.0 g)	1 × per week and 1 × per day

**Table 2:** Average larval weight gain per larvae, feed conversion ratio (FCR), feed conversion efficiency (ECI) and specific growth rate (SGR) of *Tenebrio molitor* larvae. Values are given as mean ± standard deviation, *n* = 3.

Group	LWGpL (mg)	FCR (-)	ECI (%)	SGR (% per Day)
A	16.0 ± 0.1 ^g^	5.8 ± 0.2 ^b^	17.3 ± 0.7 ^e^	2.4 ± 0.1 ^e^
B	60.5 ± 0.1 ^a^	2.3 ± 0.0 ^e^	44.4 ± 0.9 ^b^	5.1 ± 0.0 ^a^
C	8.5 ± 0.0 ^h^	8.9 ± 0.2 ^a^	11.3 ± 0.3 ^f^	1.5 ± 0.1 ^f^
D	51.9 ± 0.2 ^c^	2.3 ± 0.0 ^e^	42.8 ± 0.4 ^b^	4.8 ± 0.1 ^a^
E	31.0 ± 0.1 ^f^	2.7 ± 0.1 ^c^	36.8 ± 0.9 ^d^	3.6 ± 0.1 ^d^
F	43.8 ± 0.1 ^d^	2.3 ± 0.1 ^e^	43.8 ± 1.0 ^b^	4.4 ± 0.0 ^b^
G	38.7 ± 0.1 ^e^	2.5 ± 0.1 ^d^	39.9 ± 1.0 ^c^	4.1 ± 0.0 ^c^
H	56.2 ± 0.1 ^b^	1.8 ± 0.0 ^f^	56.1 ± 1.0 ^a^	4.9 ± 0.0 ^a^
I	55.4 ± 0.4 ^b^	1.8 ± 0.1 ^f^	55.2 ± 3.9 ^a^	4.9 ± 0.2 ^a^
J	44.7 ± 0.0 ^d^	2.2 ± 0.0 ^e^	44.6 ± 0.5 ^b^	4.4 ± 0.0 ^b^
K	60.1 ± 0.1 ^a^	2.3 ± 0.1 ^e^	42.9 ± 1.0 ^b^	5.1 ± 0.0 ^a^
L	56.6 ± 0.3 ^b^	2.5 ± 0.1 ^d^	40.2 ± 1.7 ^b^	5.0 ± 0.1 ^a^

^a–h^ Superscripts denote significant differences; *p* < 0.05; A: 75% rh, no water; B: 75% rh, 2.6 g water per day; C: 50% rh, no water; D: 50% rh, 2.6 g water per day; E: 50% rh, 3.0 g carrots per week; F: 75% rh, 2.6 g water every two days; G: 75% rh, 3.0 g carrots per week; H: 75% rh, 3.0 g carrots every two days; I: 75% rh, 3.0 g carrots per day; J: 75% rh, 10.0 g carrots per week; K: 2.6 g water per week and 3.0 g carrots per day; L: 75% rh, 2.6 g water per week and 10.0 g carrots per day.

**Table 3:** Moisture and protein content of living mealworm larvae on a fresh weight (FW) basis (%). Values are given as mean ± standard deviation, *n* = 3.

Group	Moisture (%)	Protein (% FW)
A	62.7 ± 0.7 ^c^	22.1 ± 0.1 ^b^
B	72.6 ± 0.6 ^a^	16.5 ± 0.3 ^e^
C	60.8 ± 0.1 ^d^	23.5 ± 0.1 ^a^
D	71.5 ± 0.6 ^a^	17.6 ± 0.6 ^d^
E	67.3 ± 0.5 ^b^	19.2 ± 0.3 ^c^
F	72.6 ± 2.1 ^a^	19.4 ± 0.5 ^c^
G	66.5 ± 1.2 ^b^	18.9 ± 0.5 ^c^
H	71.6 ± 0.7 ^a^	16.0 ± 0.6 ^e^
I	72.9 ± 0.5 ^a^	16.5 ± 0.1 ^e^
J	69.2 ± 2.3 ^ab^	19.5 ± 0.2 ^c^
K	70.4 ± 0.6 ^a^	18.0 ± 0.2 ^d^
L	71.5 ± 1.2 ^a^	17.4 ± 0.4 ^d^

^a–e^ Superscripts denote significant differences. A: 75% rh, no water; B: 75% rh, 2.6 g water per day; C: 50% rh, no water; D: 50% rh, 2.6 g water per day; E: 50% rh, 3.0 g carrots per week; F: 75% rh, 2.6 g water every two days; G: 75% rh, 3.0 g carrots per week; H: 75% rh, 3.0 g carrots every two days; I: 75% rh, 3.0 g carrots per day; J: 75% rh, 10.0 g carrots per week; K: 2.6 g water per week and 3.0 g carrots per day; L: 75% rh, 2.6 g water per week and 10.0 g carrots per day.

**Table 4:** Moisture and protein content in living mealworm larvae (values given in % of wet weight) for calibration and validation sets.

Statistics	Calibration Set (%)		Validation Set (%)	
	Moisture	Protein	Moisture	Protein
*n*	120	120	60	60
Mean	69.1	18.7	69.2	18.8
Minimum	60.4	16.1	61.0	16.3
Maximum	72.9	23.6	73.3	23.2
SD	3.8	2.2	3.9	2.1

SD, standard deviation.

**Table 5:** Statistics of prediction models for moisture and protein content in living mealworm larvae by NIRS (1100–2100 nm).

Item	Mathematical Treatment	No. of Latent Variables	Calibration Set		Validation Set		
			R^2^_c_	RMSEC	R^2^_p_	RMSEP	RPD
Moisture	None	7	0.922	1.084	0.910	1.171	3.33
	MC	7	0.849	1.539	0.834	1.618	2.41
	MSC	5	0.966	0.701	0.966	0.711	5.49
	1D	5	0.988	0.419	0.986	0.456	8.55
	2D	6	0.865	1.456	0.867	1.445	2.70
	MC + 1D	4	0.953	0.829	0.953	0.832	4.69
	MSC + 1D	4	0.952	0.837	0.949	0.868	4.49
Protein	None	7	0.931	0.565	0.895	0.688	3.05
	MC	7	0.856	0.821	0.808	0.932	2.25
	MSC	5	0.944	0.506	0.915	0.611	3.44
	1D	5	0.978	0.321	0.943	0.508	4.13
	2D	6	0.898	0.690	0.871	0.763	2.75
	MC + 1D	4	0.940	0.525	0.916	0.607	3.46
	MSC + 1D	4	0.913	0.631	0.892	0.683	3.08

MC: mean centering; MSC: multiple scatter correction; 1D and 2D: 1st and 2nd derivative; R^2^_c_: coefficient of determination for calibration; RMSEC: root mean square error of calibration; R^2^_p_: coefficient of determination for prediction; RMSEP: root mean square error of prediction; RPD: ratio of performance deviation.

## Data Availability

The data presented in this study are available on request from the corresponding author.

## Supplementary Materials

The following are available online at https://www.mdpi.com/article/10.3390/insects13060560/s1, Figure S1: Average NIR raw spectra (a) and preprocessed spectra (1st derivative) (b) of living mealworm larvae from all samples (*n* = 10) of all groups (*n* = 12).
