# Supplementary material for: Determination of Moisture and Protein Content in Living Mealworm Larvae (Tenebrio molitor L.) Using Near-Infrared Reflectance Spectroscopy (NIRS)
Source: Insects. 2022 Jun 20;13(6):560. doi: 10.3390/insects13060560 (PMC9224910; doi:10.3390/insects13060560)
Supplement: Supplementary file 1 [file insects-13-00560-s001.zip › insects-1746006-supplementary.pdf]

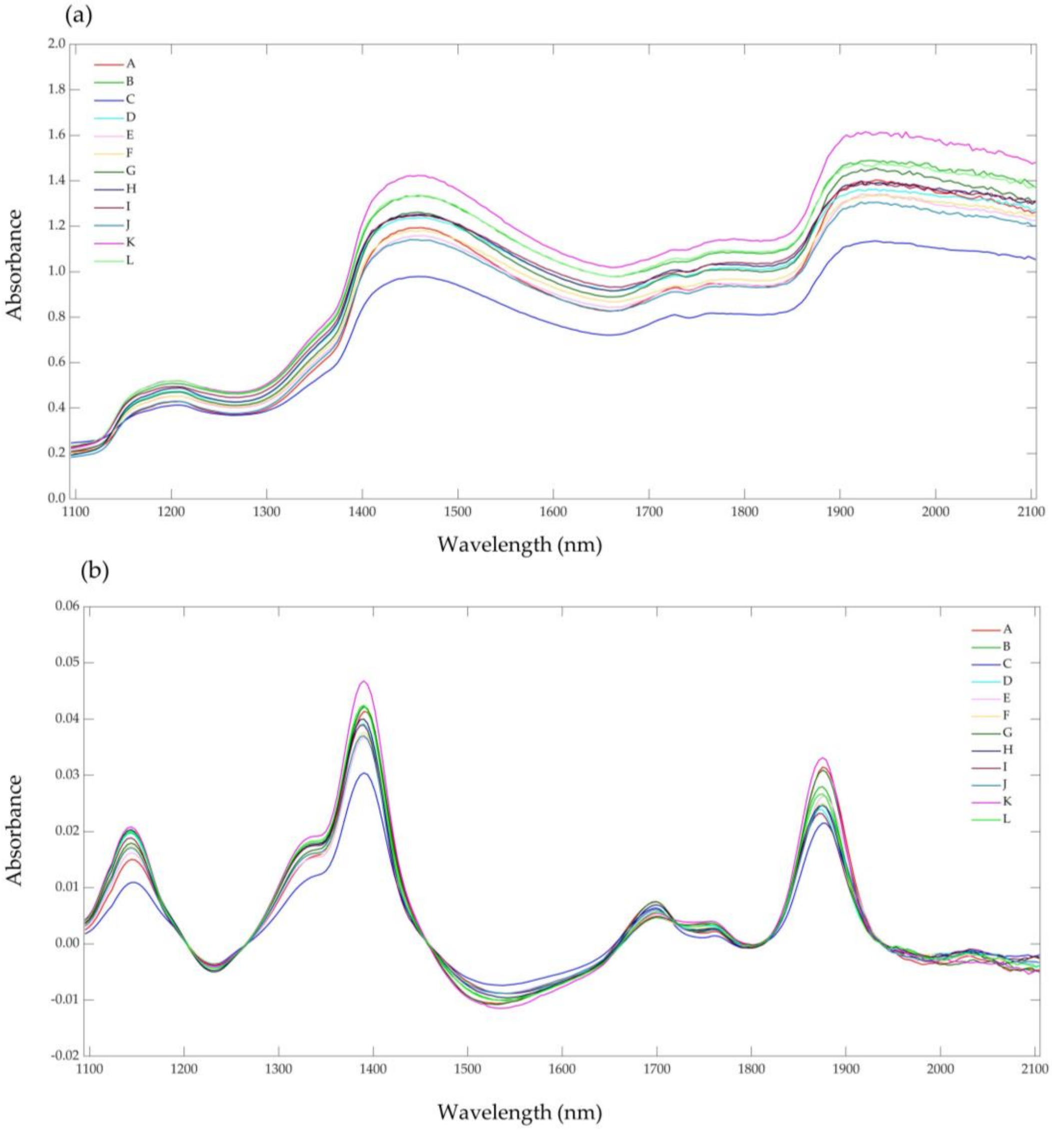

Figure S1. Average NIR raw spectra (a) and preprocessed spectra (firstderivative) (b) of living mealworm larvae from all samples (n = 10) of all groups (n = 12). A: 75% rh, no water; B: 75% rh, 2.6 g water per day; C: 50% rh, no water; D: 50% rh, 2.6 g water per day; E: 50% rh, 3.0 g carrots per week; F: 75% rh, 2.6 g water every two days; G: 75% rh, 3.0 g carrots per week; H: 75% rh, 3.0 g carrots every two days; I: 75% rh, 3.0 g carrots per day; J: 75% rh, 10.0 g carrots per week; K: 2.6 g water per week and 3.0 g carrots per day; L: 75% rh, 2.6 g water per week and 10.0 g carrots per day.
